# Spatio-temporal analysis of the genetic diversity and complexity of *Plasmodium falciparum* infections in Kedougou, southeastern Senegal

**DOI:** 10.1186/s13071-017-1976-0

**Published:** 2017-01-19

**Authors:** Makhtar Niang, Laty G. Thiam, Cheikh Loucoubar, Abdourahmane Sow, Bacary D. Sadio, Mawlouth Diallo, Amadou A. Sall, Aissatou Toure-Balde

**Affiliations:** 1 Institut Pasteur Dakar, Immunology Unit, 36 Avenue Pasteur, BP 220 Dakar, Senegal; 2 Department of Animal Biology, Cheikh Anta Diop University of Dakar, Dakar, Senegal; 3 Institut Pasteur Dakar, Biostatistics, Bioinformatics and Modeling Group, 36 Avenue Pasteur, BP 220 Dakar, Senegal; 4 Institut Pasteur Dakar, Arbovirus and Viral Hemorrhagic Fevers Unit, 36 Avenue Pasteur, BP 220 Dakar, Senegal; 5 Institut Pasteur Dakar, Medical Entomology Unit, 36 Avenue Pasteur, BP 220 Dakar, Senegal

**Keywords:** Malaria, *Plasmodium falciparum*, Transmission, Diversity, Kedougou

## Abstract

**Background:**

Genetic analyses of the malaria parasite population and its temporal and spatial dynamics could provide an assessment of the effectiveness of disease control strategies. The genetic diversity of *Plasmodium falciparum* has been poorly documented in Senegal, and limited data are available from the Kedougou Region. This study examines the spatial and temporal variation of the genetic diversity and complexity of *P. falciparum* infections in acute febrile patients in Kedougou, southeastern Senegal. A total of 263 sera from patients presenting with acute febrile illness and attending Kedougou health facilities between July 2009 and July 2013 were obtained from a collection established as part of arbovirus surveillance in Kedougou. Samples identified as *P. falciparum* by nested PCR were characterized for their genetic diversity and complexity using *msp-1* and *msp-2* polymorphic markers.

**Results:**

Samples containing only *P. falciparum* accounted for 60.83% (160/263) of the examined samples. All three *msp-1* allelic families (K1, MAD20 and RO33) and two *msp-2* allelic families (FC27 and 3D7) were detected in all villages investigated over the 5-year collection period. The average genotype per allelic family was comparable between villages. Frequencies of *msp-1* and *msp-2* allelic types showed no correlation with age (Fisher’s exact test, *P* = 0.59) or gender (Fisher’s exact test, *P* = 0.973), and were similarly distributed throughout the 5-year sampling period (Fisher’s exact test, *P* = 0.412) and across villages (Fisher’s exact test, *P* = 0.866). Mean multiplicity of infection (MOI) for both *msp-1* and *msp-2* was highest in Kedougou village (2.25 and 2.21, respectively) and among younger patients aged ≤ 15 years (2.12 and 2.00, respectively). The mean MOI was highest in 2009 and decreased progressively onward.

**Conclusion:**

Characterization of the genetic diversity and complexity of *P. falciparum* infections in Kedougou revealed no spatio-temporal variation in the genetic diversity of *P. falciparum* isolates. However, mean MOI varied with time of sera collection and decreased over the course of the study (July 2009 to July 2013). This suggests a slow progressive decrease of malaria transmission intensity in Kedougou Region despite the limited impact of preventive and control measures implemented by the National Malaria Control Programme on malaria morbidity and mortality.

## Background

According to the World Health Organization (WHO), the incidence of malaria and associated mortality respectively decreased by 30 and 47% globally between 2000 and 2013 [1]. Despite this significant achievement resulting from coordinated malaria preventive and control interventions, an estimated 198 million cases and about 584,000 deaths still occurred worldwide in 2014 [1].

In Senegal, malaria remains a public health concern even though malaria control interventions have successfully reduced the incidence rate nationwide over the last decade [2]. Nonetheless, the situation is particularly worrying in the Southeastern part of the country due to limited impact of applied preventive and control measures [2]. In Kedougou, a region situated in southeastern Senegal, malaria due to *Plasmodium falciparum* remains highly prevalent [2, 3], particularly during the transmission season that coincides with the rainy season from July to November. In 2014, the National Malaria Control Program (NMCP) reported 25.55% of malaria incidence of which 2.73% turned into severe disease [2].

Extensive parasite genetic diversity and routine carriage of multiple parasite genotypes by malaria-infected individuals are generally observed in areas of intense malaria transmission [4, 5]. The genetic diversity of *P. falciparum* parasites is therefore an indicator of malaria transmission intensity, thus serving as a tool to evaluate the effectiveness of malaria control interventions.

The merozoite surface protein-1 (*msp-1*) and -2 (*msp-2*) are polymorphic antigenic markers that have been extensively used for genetic characterization of parasite populations in malaria endemic areas but have also served to distinguish new from recrudescent infections during anti-malarial drug trials and efficacy studies [6–8].

Despite previous investigations of the genetic diversity of *P. falciparum* in Senegal [9–12], further detailed studies are needed particularly in Kedougou Region where malaria transmission is still active. The present study examined the genetic diversity and complexity or multiplicity of infection (MOI) of *P. falciparum* infections among febrile patients in Kedougou Region. The spatial and temporal distributions of *msp-1* and *msp-2* allelic families of *P. falciparum* isolates were also analyzed.

## Methods

### Population and study design

**Fig. 1:**
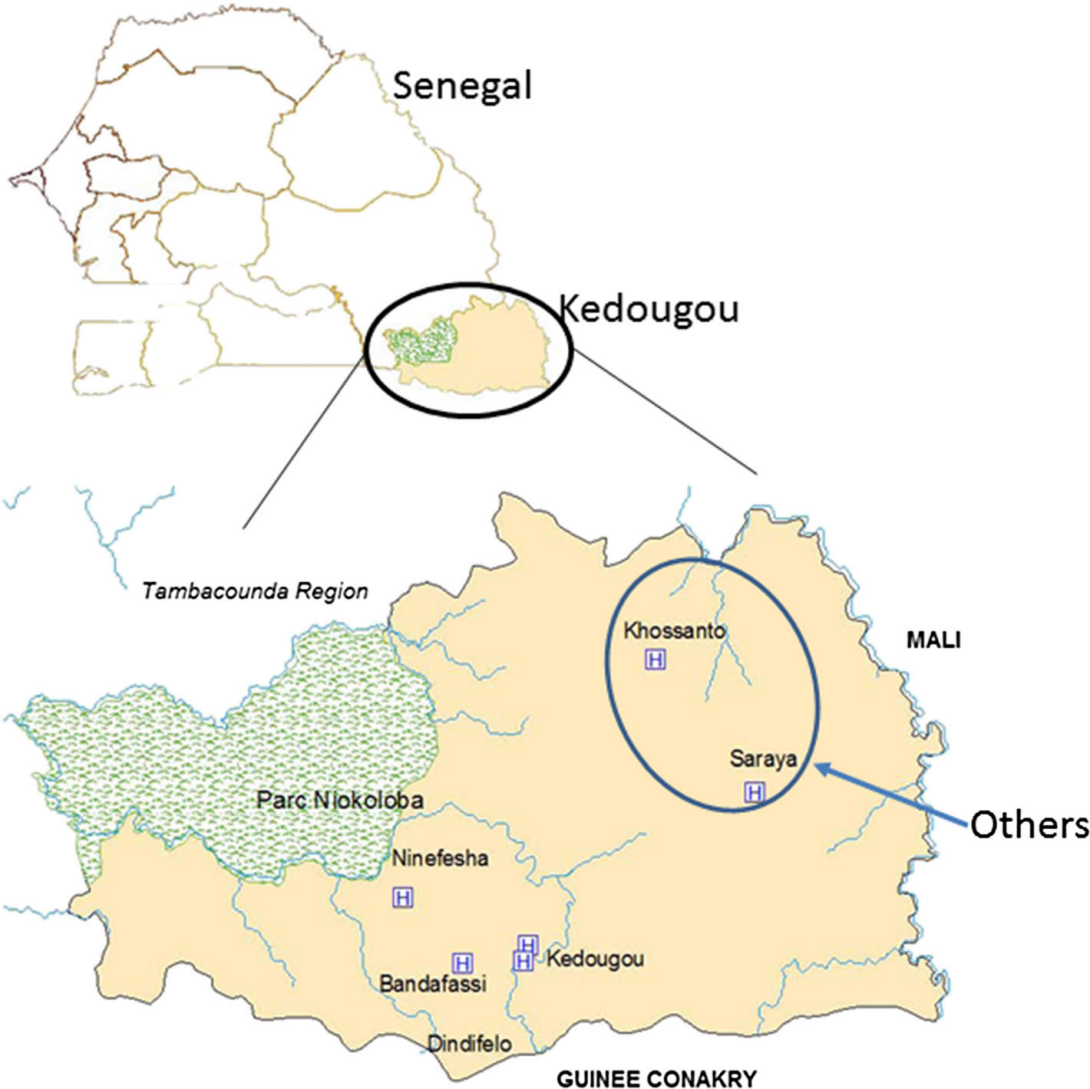
Map of Kedougou Region showing village of origin of samples. The three major villages (Kedougou, Bandafassi and Ninefesha) where most samples originated are shown, the remaining villages grouped under the term “others” are also shown

The samples used in this study originated from Kedougou Region (Fig. 1), southeastern Senegal. A detailed description of the study area (climate, rainfall, landscape, fauna, population) has been provided elsewhere [3, 13, 14]. The Kedougou Region borders Guinea, Mali and Gambia between isohyets 1200 and 1300 mm. A total of 263 sera from patients with acute febrile illness (AFI) visiting healthcare facilities (Bandafassi, Kedougou, Ninefesha, Saraya, Khossanto) in Kedougou Region between July 2009 and July 2013 were obtained from a collection established during arbovirus surveillance in Kedougou Region [14]. Due to the similar clinical presentation between malaria and arboviral infection, malaria diagnostic screening was systematically conducted to differentiate arboviral to malaria disease.

AFI was defined as “any patient older than 1 year with a fever (temperature > 38 °C) lasting for less than 2 weeks, exhibiting two or more of the following symptoms: headache, myalgia, eye pain, arthralgia, cough, nausea/vomiting, diarrhea, jaundice, bleeding and/or neurological signs”.

Sera were investigated for presence of only *P. falciparum* and subsequently analyzed for genetic diversity, temporal and spatial distribution of allelic families and complexity of malaria infection.

### Characterization of malaria parasite species

To ensure that patients were infected with only *P. falciparum*, all 263 sera were subjected to *Plasmodium* species characterization following genomic DNA (gDNA) isolation using QIamp DNA Mini Kit (Qiagen, Hilden, Germany) as reported by others [15, 16]. DNA extracted from blood samples of known microscopically confirmed *P. falciparum*, *P. malariae*, *P. vivax* and *P. ovale*-infected patients were used as positive controls [3] to discriminate *P. falciparum-*positive samples from other *Plasmodium*-infected samples.

Qualitative detection of *Plasmodium* parasite DNA was based on a nested PCR approach with primers targeting the *Plasmodium* spp. 18S small subunit ribosomal RNA gene (18S ssrDNA) as described previously [17]. The primary and nested PCR reactions were performed using the GoTaq Green Master Mix protocol (Promega, Madison, USA), according to the manufacturer’s recommendations, and amplification conditions were as described previously [3]. Nested PCR results were scored as a categorical variable (presence *versus* absence of amplification).

### Genotyping of *Plasmodium falciparum* isolates

Genetic characterization of *P. falciparum* isolates was carried out by nested PCR amplification of the two highly polymorphic regions of *msp-1* (block 2) and *msp-2* (block 3) genes as described previously [18, 19]. Primer sequences used for amplification of the three allelic families of *msp-1* (K1, MAD20 and RO33) and two allelic families of *msp-2* (FC27 and 3D7) have been reported elsewhere [11, 19, 20]. An initial amplification of the outer regions of the two genes was followed by a nested-PCR with allelic family specific primer pairs. In the primary PCR reaction, 2 μl of gDNA served as template while in the nested reaction, 1 μl of the outer PCR product was used as template. Amplification products were separated by electrophoresis on 1.5% agarose gel, and the fragments were visualized using ethidium bromide under UV light. The sizes of amplified products were determined using a molecular weight marker.

The frequency of each *msp* allelic family was calculated as the number of *P. falciparum* isolates containing at least one allele from that family out of the total number of samples. The multiplicity of infection (MOI) or number of genotypes per infection was calculated by dividing the total number of fragments detected in *msp-1* or *msp-2* by the number of samples positive for the same marker [21, 22].

### Statistical analysis

Data were analyzed using R statistical software version 3.1.1 (2014-07-10) [23]. Distributions of age and sex between villages were compared using Kruskall-Wallis rank sum test. All comparisons between frequencies were performed using Fisher’s exact test. Comparisons were considered statistically significant when *P*-values were less than the Bonferroni-corrected threshold.

## Results

### Characteristics of the study population

All 263 serum samples were screened for the presence of *Plasmodium* parasite DNA and characterization of *Plasmodium* species [3]. Samples containing only *P. falciparum* accounted for 60.83% (160/263) and were further characterized for genetic diversity and MOI using *msp-1* and *msp-2* polymorphic markers. The demographic characteristics of the 160 *P. falciparum*-positive patients are summarized in Table 1.

**Table 1:** Baseline characteristics of the study population

	Kedougou *n* (%)	Bandafassi *n* (%)	Ninefesha *n* (%)	Others *n* (%)	Total *N* (%)	*P*-value
	47 (29.37)	72 (45)	18 (11.25)	23 (14.37)	160 (100)	
Age						
Mean (yrs)	22	15	14	23	18	0.0012
Range (yrs)	7–60	1–65	2–52	1–59	1–65	
Sex						
Male	26 (55)	37 (51)	8 (44)	14 (61)	86 (53.75)	
Female	21 (45)	35 (49)	10 (56)	9 (39)	74 (45.25)	
Ratio (M/F)	1.23	1.05	0.8	1.55	1.16	0.743
Age group						
≤ 15 years	14 (29.79)	47 (65.28)	12 (66.66)	9 (39.13)	82 (51.25)	
> 15 years	33 (70.21)	25 (34.72)	6 (33.33)	14 (60.87)	78 (48.75)	0.001

Patients ranged in age from one to 65 years old and the majority (45%) originated from the village of Bandafassi (Table 1). The mean age of patients was 18 years but varied significantly between villages (Kruskal-Wallis H-test: *χ* ^2^ = 15.901, *df*  =  3, *P* = 0.0012) (Table 1). Most patients (51.25%) were ≤ 15 years despite variations between villages (Fisher's exact test, *P* = 0.001). Among the 160 *P. falciparum*-infected patients, 53.75% (86/160) were male and 45.25% (74/160) were female. The sex ratio (M/F) varied between villages and was in favor of males except for Ninefesha (Table 1), although this difference was not significant (Kruskall Wallis rank test, *P* = 0.743).

### Genetic diversity of *Plasmodium falciparum* isolates

All three allelic families of *msp-1* (K1, MAD20 and RO33) and two of *msp-2* (3D7 and FC27) were detected among the 160 *P. falciparum* positive isolates genotyped in this study.

Of the detected *msp-1* genotypes, 41.45% (182/439) belonged to the MAD20 allelic family while K1 and RO33 allelic families represented 34.16% (150/439) and 24.37% (107/439), respectively (Table 2). The 3D7 allelic type was the predominant *msp-2* genotype representing 77.89% (215/276) of detected *msp-2* genotypes while the FC27 allelic type represented only 22.10% (61/276) of *msp-2* genotypes (Table 2).

**Table 2:** Total and mean number of genotypes per allelic family

Village	*msp-1*			Total	*msp-2*		Total
	K1	MAD20	RO33		FC27	3D7	
Kedougou	47 (1.00)	51 (1.08)	37 (0.78)	135	16 (0.34)	46 (0.97)	62
Bandafassi	65 (0.90)	85 (1.18)	42 (0.58)	192	34 (0.47)	113 (1.56)	147
Ninefesha	17 (0.94)	18 (1.00)	8 (0.44)	43	6 (0.33)	32 (1.77)	38
Others	21 (0.91)	28 (1.21)	20 (0.86)	69	5 (0.21)	21 (1.04)	29
Total	150	182	107	439	61	215	276

Although the total number of genotypes was highest in *P. falciparum* isolates obtained from Bandafassi (Table 2), the mean number of genotypes per allelic family was comparable between villages.

### Frequency of *msp-1* and *msp-2* allelic families

**Fig. 2:**
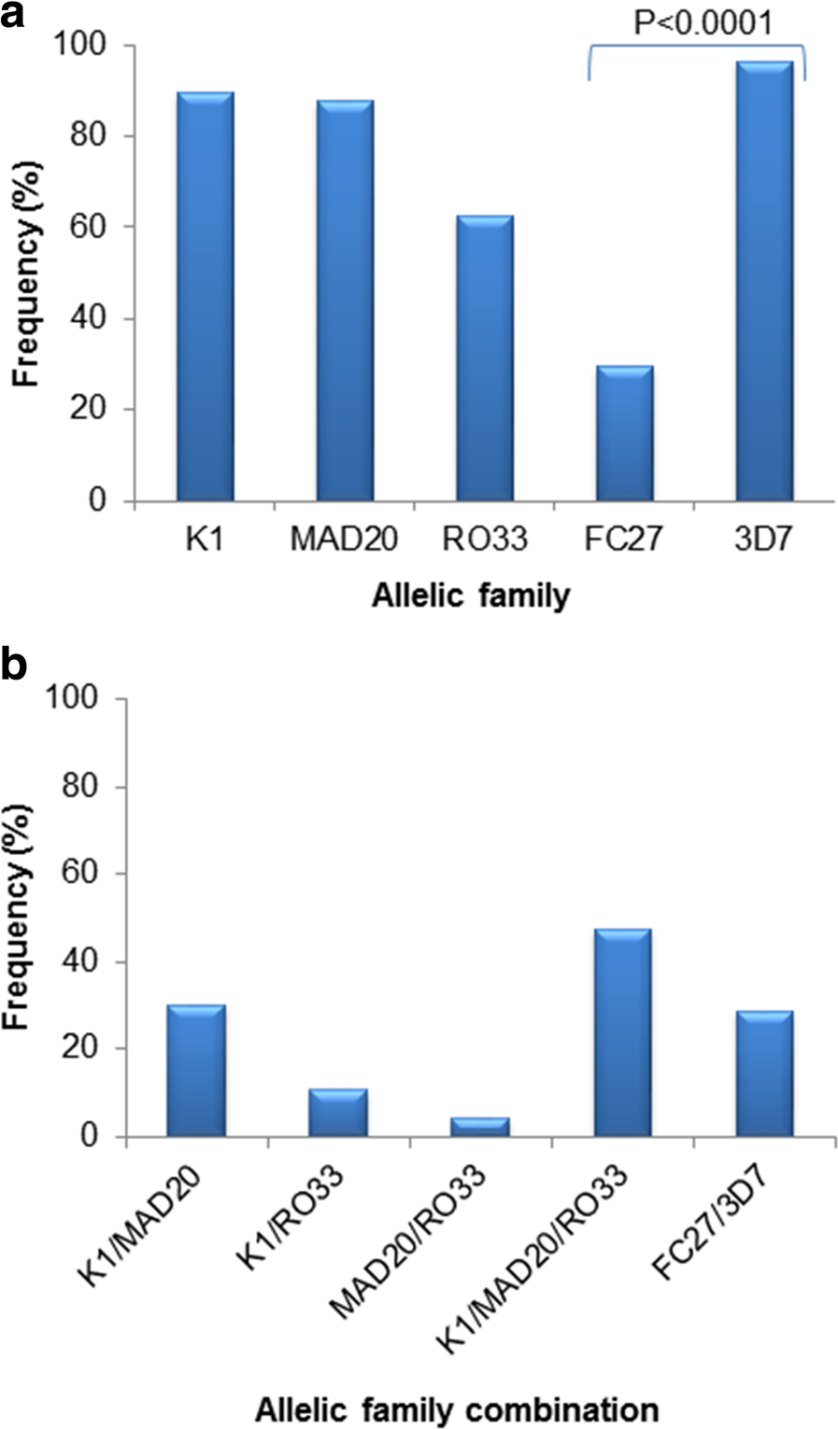
Frequency of unique (**a**) and combined (**b**) *msp-1* and *msp-2* allelic families of *Plasmodium falciparum* isolates from Kedougou Region. Alleles of the *msp-1* and *msp-2* genes were classified by the presence of fragment resulting from the PCR amplifications with allele specific primers. Pairwise Fisher’s exact tests between allelic families showed only significant difference between FC27 and 3D7

All individual *msp-1* and *msp-2* allelic families, dimorphic combinations of *msp-1* (K1/MAD20, K1/RO33 and MAD20/RO33) and *msp-2* (FC27/3D7), and trimorphic combination of *msp-1* (K1/MAD20/RO33) were observed at varying proportions in *P. falciparum* isolates from Kedougou Region (Fig. 2).

Concerning the *msp-1* locus, the K1 and MAD20 allelic families were the most represented and were identified in 89.37% (143/160) and 87.5% (140/160) of samples, respectively, while the RO33 allelic family was present in 62.5% (100/160) of samples (Fig. 2a). Dimorphic *msp*-1 allelic combinations accounted for 30% (48/160), 10.62% (17/160) and 4.37% (7/160) of *msp-1* positive samples for K1/MAD20, K1/RO33 and MAD20/RO33, respectively, while the percentage of samples carrying the three *msp-1* allelic families (K1/MAD20/RO33) was 47.5% (76/160) (Fig. 2b).

With respect to *msp*-2, the frequency of samples with only the 3D7 allelic family [96.25% (154/160)] was found to be significantly higher (Fisher’s exact test, *P* < 0.001) than those harboring only the FC27 allelic family [29.37% (47/160)] (Fig. 2a). Dimorphic FC27/3D7 allelic combination was identified in 28.75% (46/160) of samples (Fig. 2b).

### Frequency of *msp-1* and *msp-2* allelic types with respect to age and gender

**Fig. 3:**
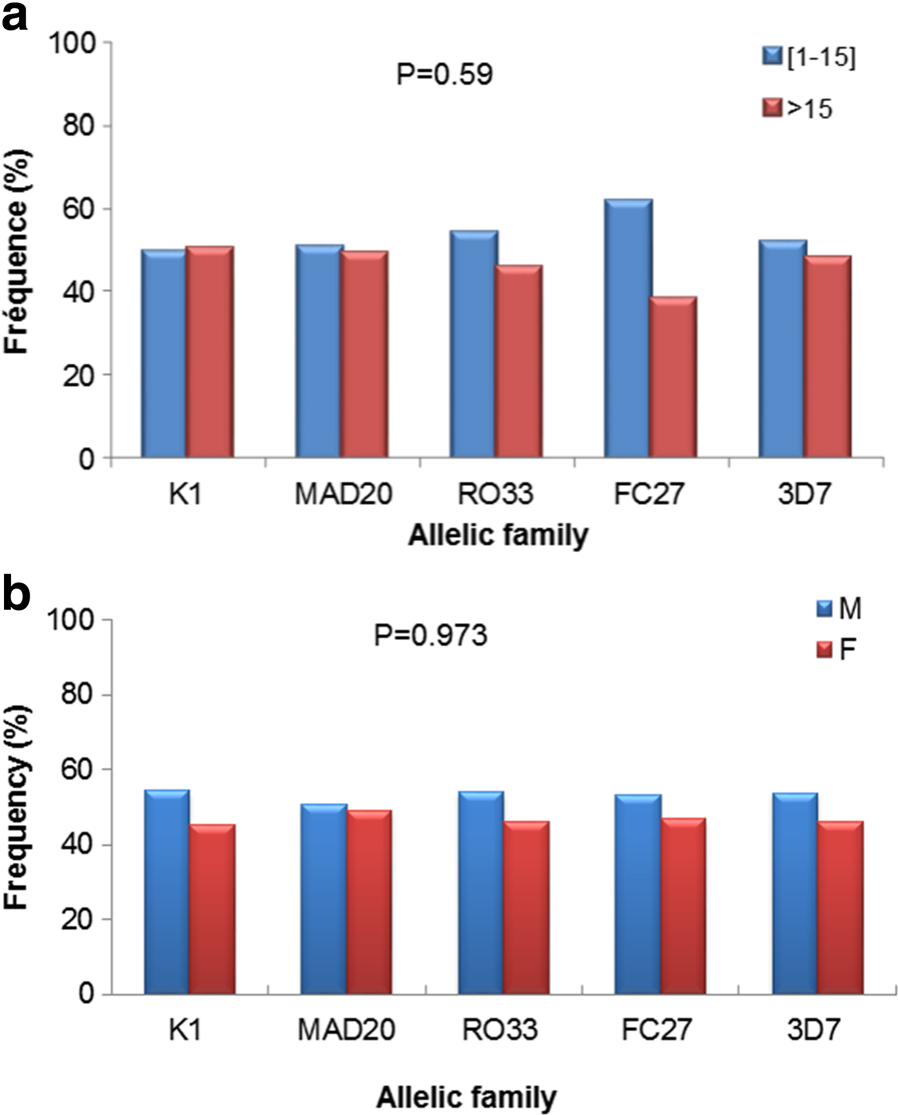
Distribution of frequencies of *msp-1* and *msp-2* allelic families according to age group (**a**) and gender (**b**). Frequencies were determined as the percentage of individuals within a given age group or gender among the positive samples. Statistical differences were determined using Fisher’s exact test

The frequencies of *msp-1* and *msp-2* allelic types were categorized within two arbitrarily defined age groups: ≤ 15 years and > 15 years (Table 1). Frequencies of the three *msp-1* allelic types (K1, MAD20 and RO33) and two *msp-2* allelic types (FC27 and 3D7) were similarly distributed with no significant differences between age groups (Fisher’s exact test, *P* = 0.59) (Fig. 3a). Within a given age group, frequencies of the three *msp-1* allelic types and the two *msp-2* allelic types were also comparable.

With respect to gender, frequencies of *msp-1* and *msp-2* allelic families showed similar distribution between males and females with no significant difference observed (Fisher’s exact test, *P* = 0.973). The frequencies of all *msp-1* and *msp-2* allelic families were also comparable within a given gender (Fig. 3b).

### Temporal and spatial distribution of *msp-1* and *msp-2* allelic families in Kedougou Region

**Fig. 4:**
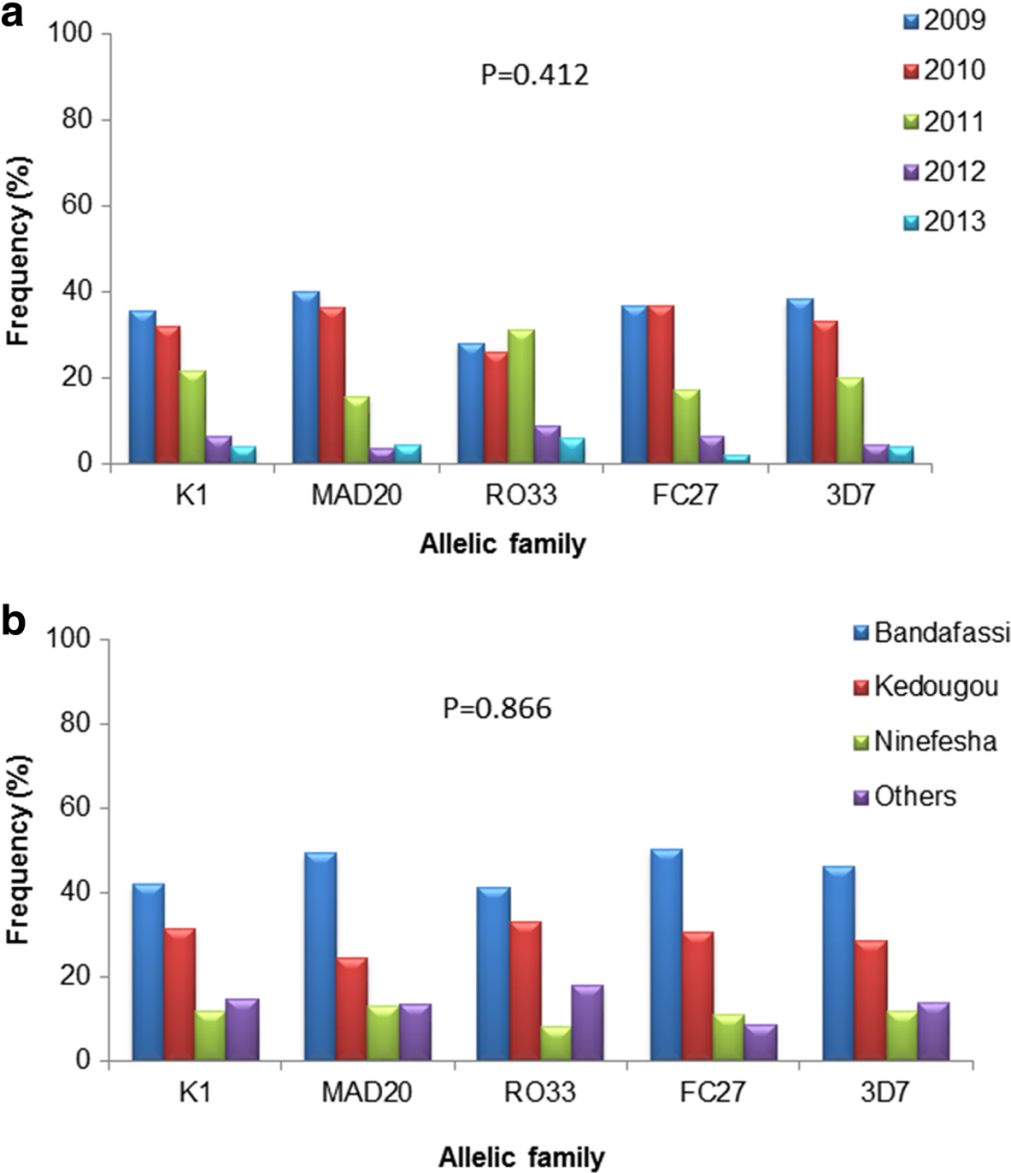
Temporal (**a**) and spatial (**b**) distribution of *msp-1* and *msp-2* allelic families in Kedougou Region. Frequencies of allelic families according to period (temporal distribution) or village (spatial distribution) were calculated as the percentage of samples positive for a given allelic family for a period or village out of the total positive samples. Statistical differences were determined using Fisher’s exact test

Analysis of the spatio-temporal distribution of all *msp-1* and *msp-2* allelic families of *P. falciparum* isolates from 2009 to 2013 showed no significant difference (Fisher’s exact test, *P* = 0.412) throughout the 5-year sampling period (Fig. 4a). Allelic frequencies were highest in 2009 and decreased progressively up to 2013 except for RO33 in 2011 (Fig. 4a). The distribution of allelic frequencies was also similar (Fisher’s exact test, *P* = 0.866) between villages with a consistent low frequency of all allelic types in Ninefesha (Fig. 4b), probably related to the lower number of samples from this village.

### Mean multiplicity of infection in relation to village, age and sampling period

Mean MOI was compared between villages (Kedougou, Bandafassi, Ninefesha and others), age groups (≤ 15 years and > 15 years), and sampling periods (2009 to 2013). The mean MOI for both *msp-1* and *msp-2* was found to be higher in *P. falciparum* isolates from patients from Kedougou village (MOI = 2.25 and 2.21), in patients aged ≤ 15 years (MOI = 2.12 and 2.00), and those diagnosed in 2009 (MOI = 2.21 and 2.50) than *P. falciparum* isolates originating from others villages, patients aged older than 15 years, and those diagnosed from 2010 onward, respectively (Table 3). Overall, the mean MOI varied between villages for both loci and decreased progressively with increasing age and year of sample collection for both *msp-1* and *msp-2* loci (Table 2).

**Table 3:** Mean multiplicity of infection (MOI) among the study participants with respect to sample origin, age and period of collection

	No. of samples	Mean MOI	
		*msp-1*	*msp-2*
Village			
Kedougou	47	2.25	2.21
Bandafassi	72	1.73	2.06
Ninefesha	18	1.79	1.82
Others	23	1.76	1.62
Age group			
≤ 15 years	82	2.12	2.00
> 15 years	78	1.64	1.84
Year			
2009	61	2.21	2.50
2010	51	2.05	2.25
2011	32	1.90	1.95
2012	9	1.65	1.70
2013	7	1.60	1.20

## Discussion

The present study provides information about the spatial and temporal dynamics of the *P. falciparum* parasite population among febrile patients in Kedougou Region. This bridges an important gap in the understanding of the molecular characteristics of the parasite population in this region where *Plasmodium* transmission is still active and could inform efforts to monitor and control *Plasmodium* transmission in this region.

All *msp-1* and *msp-2* allelic families were represented in all villages in Kedougou Region where samples were collected and elevated frequencies of *msp-1* and *msp-2* genotypes were reported with MAD20 and 3D7 being the most prevalent, particularly in Bandafassi and Kedougou villages. However, the comparable average number of genotypes for each locus between villages suggested that the large number of genotypes in Bandafassi potentially reflects an elevated number of samples rather than higher genetic diversity of *P. falciparum* isolates in this village.

FC27 allelic type has been shown to be more prevalent in asymptomatic rather than symptomatic *Plasmodium*-infected individuals [24], suggesting a lower risk of developing symptomatic malaria with increasing carriage of isolates belonging to the FC27 allelic family. Such observations are supported by previous studies in Senegal [5, 13] and Nigeria [25] but contrast with data from Benin and Gabon [26, 27], emphasizing the need for multicentric studies using *P. falciparum* isolates from both asymptomatic and symptomatic individuals to comprehensively establish the potential relationship between specific allele carriage and disease outcome. High to moderate mean MOI was observed among *P. falciparum* isolates across all the studied villages with the highest mean MOI being observed in Kedougou village. A high MOI is a common feature in most malaria hyperendemic areas [28–30] and has been directly linked with malaria transmission intensity [22, 28, 30]. Thus, the slow decrease of the mean MOI for both *msp-1* and *msp-2* loci from 2009 to 2013 in Kedougou Region could suggest a progressive decrease of *Plasmodium* transmission although a lack of data prior major interventions by the NMCP in Kedougou Region does not allow for a thorough comparison.

To date, only a few studies have applied genetic surveillance tools to evaluate *Plasmodium* population structure in relation to changing malaria transmission in Senegal, thus precluding a meaningful comparison with the situation observed in Kedougou Region. The application of molecular barcoding genomic surveillance on *P. falciparum* parasites collected from Thies region (West Senegal) between 2006 and 2013 revealed reduced genetic diversity of *P. falciparum* population along with a decline of *Plasmodium* transmission from 2006 to 2010, a period transcending the introduction of insecticide-treated bednets in 2008 [31]. The mass distribution of insecticide-treated bednets in malaria-endemic countries was demonstrated to be a key player in the dramatic decline of *Plasmodium* transmission documented in Senegal [32, 33] and elsewhere [34].

The finding that MOI decreased with age supports studies conducted in other countries [35, 36] that have shown lower MOI in those aged 15 and above. The existence of a positive association between high childhood MOI and low adulthood MOI is in keeping with the concept that exposure to multiple strains of the parasite in early life is necessary to produce immunity in adults. Conflicting findings indicating a greater MOI in older individuals have also been reported [29, 37]. The limited studies on *P. falciparum* genetic diversity conducted in Senegal failed to establish a link between MOI and age groups [10–12], thus depicting the complex nature of such an association. The lack of an association between MOI and age groups as reported in above-mentioned studies suggests that the MOI is not directly related to the period of acquisition of immunity, but rather reflects the exposure of subjects to malaria in an endemic area. The decrease of MOI over time despite the limited impact of control measures suggested a selection of clonal parasites populations, thus increasing the chance of an individual being re-infected with the same allele.

## Conclusions

This study reports the spatial and temporal variation of the genetic diversity and complexity of *P. falciparum* infections in acute febrile patients in Kedougou, a Senegalese region with active malaria transmission. The findings revealed that *P. falciparum* isolates of Kedougou Region are highly diverse with limited spatio-temporal variation in the region. However, MOI decreased over time, which may be an indication of a progressive reduction in *Plasmodium* transmission in the region despite the limited impact of control measures on malaria morbidity and mortality in the area.

Additional studies that examine the dynamics of the genetic diversity of *P. falciparum* parasite populations, considering factors such as the malaria transmission intensity based on entomological inoculation rates and the immune status of *P. falciparum*-infected individuals would tremendously improve our understanding of *P. falciparum* parasite diversity in Kedougou Region and contribute to guide malaria interventions.

## Abbreviations

AFI: Acute febrile illness
MOI: Multiplicity of infection
NMCP: National Malaria Control Programme
WHO: World Health Organization
